# AnnoDash, a clinical terminology annotation dashboard

**DOI:** 10.1093/jamiaopen/ooad046

**Published:** 2023-07-08

**Authors:** Justin Xu, Mjaye Mazwi, Alistair E W Johnson

**Affiliations:** Child Health Evaluative Sciences, The Hospital for Sick Children, Toronto, Ontario, Canada; Department of Critical Care Medicine, The Hospital for Sick Children, Toronto, Ontario, Canada; Child Health Evaluative Sciences, The Hospital for Sick Children, Toronto, Ontario, Canada

**Keywords:** clinical concepts, ontology, annotation, natural language processing, software

## Abstract

**Background:**

Standard ontologies are critical for interoperability and multisite analyses of health data. Nevertheless, mapping concepts to ontologies is often done with generic tools and is labor-intensive. Contextualizing candidate concepts within source data is also done in an ad hoc manner.

**Methods and Results:**

We present AnnoDash, a flexible dashboard to support annotation of concepts with terms from a given ontology. Text-based similarity is used to identify likely matches, and large language models are used to improve ontology ranking. A convenient interface is provided to visualize observations associated with a concept, supporting the disambiguation of vague concept descriptions. Time-series plots contrast the concept with known clinical measurements. We evaluated the dashboard qualitatively against several ontologies (SNOMED CT, LOINC, etc.) by using MIMIC-IV measurements. The dashboard is web-based and step-by-step instructions for deployment are provided, simplifying usage for nontechnical audiences. The modular code structure enables users to extend upon components, including improving similarity scoring, constructing new plots, or configuring new ontologies.

**Conclusion:**

AnnoDash, an improved clinical terminology annotation tool, can facilitate data harmonizing by promoting mapping of clinical data. AnnoDash is freely available at https://github.com/justin13601/AnnoDash (https://doi.org/10.5281/zenodo.8043943).

## INTRODUCTION

Digitized electronic health record (EHR) data typically includes a wide array of heterogeneous concepts which are useful in monitoring and evaluating patient health status.^1^^,^^2^ Aggregated multi-institutional EHR data has the potential to provide new insight into health and clinical care.^3^^,^^4^ Integration of data across institutes is a challenging endeavor as the underlying digital infrastructure is often locally customized.^5–7^ A common manifestation of this phenomenon relates to the storage of clinical concepts using locally specific coded terms. For example, rather than storing the phrase “Systolic Blood Pressure” with every associated measurement, EHR systems will create a coded term to identify these measurements and use a reference table when the user requires a human interpretable description of the code. Multisite research requires harmonization of these locally created concept codes which correspond to the same underlying phenomena, a process supported by the significant effort invested in the creation of standard ontologies.^8^

Ontologies have become increasingly important as they allow for consistent and accurate representation of medical concepts, providing a common language for professionals and researchers in medical informatics. Systematized Nomenclature of Medicine Clinical Terms (SNOMED CT), maintained by the nonprofit organization SNOMED International, and Logical Observation Identifiers Names and Codes (LOINC), developed by the Regenstrief Institute, are 2 such clinical ontologies. SNOMED CT and LOINC provide a standardized vocabulary for representing and exchanging medical knowledge by defining identifier codes for medical concepts.^9^^,^^10^

Previous work has aimed to automatically map concepts to standard ontologies, for example, by mapping laboratory tests to LOINC codes using machine learning.^11^ Despite these efforts, assignment of ontology codes to clinical concepts is still often done manually, relying on heuristics for matching free-text descriptions associated with the concept. Tools to support the mapping process include Regenstrief LOINC Mapping Assistant (RELMA), now discontinued, and Usagi, designed by the Observational Health Data Sciences and Informatics (OHDSI) team.^12^ Usagi uses Apache Lucene with custom string processing to provide the most likely candidate matches for a given concept from the Observational Medical Outcomes Partnership (OMOP) vocabulary, which is a versioned combination of various other standard ontologies and may lag behind its constituent ontology systems. Usagi has a number of advantages including high performance and a well-structured tutorial. However, it contains several customizations designed for mapping to OMOP. Furthermore, although related string fields can be included when loading in source concepts, Usagi does not provide a mechanism for reviewing data in aggregate associated with a concept or within individual patient contexts. To disambiguate concepts, the typical approach would be to search for the concept code within the EHR and review the context in which it is used. This process is time-consuming and often provides an incomplete picture of all the possible uses of the concept.

Here we present a flexible and customizable dashboard for annotation of clinical terminology. The objective of the dashboard is threefold: (1) to integrate data visualization options for the viewing of clinical concepts in the context of patient records, (2) to optimize the speed and accuracy of terminology mapping with search suggestions based on natural language processing and re-ranking using large language models (LLMs), and (3) to maintain a modular code structure, allowing users to easily incorporate extensions such as machine learning-powered plugins or other search algorithms.

## METHODOLOGY

The dashboard was developed based on the Plotly Dash library in Python.^13^^,^^14^ Plotly Dash allows for the creation of interactive, web-based plots by combining common web components which enable the display or manipulation of information. The dashboard has 3 areas of interest: the concept pane (top left), the visualization pane (top right), and the search pane (bottom).

The concept pane lists the terms which require annotation. In this pane, the user is able to navigate through the source concepts for annotation, select the target ontology, and review selected ontology terms.

The visualization pane in the top right provides plots summarizing quantitative measurements associated with the selected source concept. A distribution plot is created for each concept to visualize observed values. For numerical concepts, the dashboard generates a count distribution for the entire range of values, with the numerically lowest observation on the left. For textual concepts, the dashboard generates a bar graph and plots the counts for each observed text value in alphabetical order. These aggregated plots give an overview of concepts and provide context beyond their associated textual descriptions. Tabs above the figure allow switching to individual time-series plots associating measurements for the concept with time, as well as with measured data of already known clinical concepts.

Finally, the bottom pane enables searching and filtering of the ontology terms. We experimented with multiple approaches for suggesting concepts from the target ontology based upon string similarity to textual descriptions of source codes, including fuzzy string matching and vector search over embeddings and indices.

Ontology terms are loaded into a relational database (SQLite by default). Instructions and scripts for loading LOINC version 2.73, SNOMED CT International Edition update 07/31/2022, ICD-10 Clinical Modification 2022, and OMOP version 5 are available. Other target ontology systems may be added through the generation of an associated SQLite database using the provided scripts.

In addition to standalone search, we have implemented support for re-ranking of suggested terms using LLMs. As LLMs currently do not have sufficient context to parse entire ontology systems, we have followed the paradigm of high sensitivity retrieval followed by high performance LLM re-ranking. By default, users have the option to use OpenAI’s GPT-3.5 model or CohereAI’s re-ranking API endpoint to support their workflow. The GPT-3.5 prompts also enable the use of quantitative measurements associated with the source concept to improve rankings. As these features incur additional monetary costs, they have been implemented as another entirely standalone module which is disabled by default. As with the rest of the dashboard, users with more technical experience may configure the LLM used.

**Figure 1.**
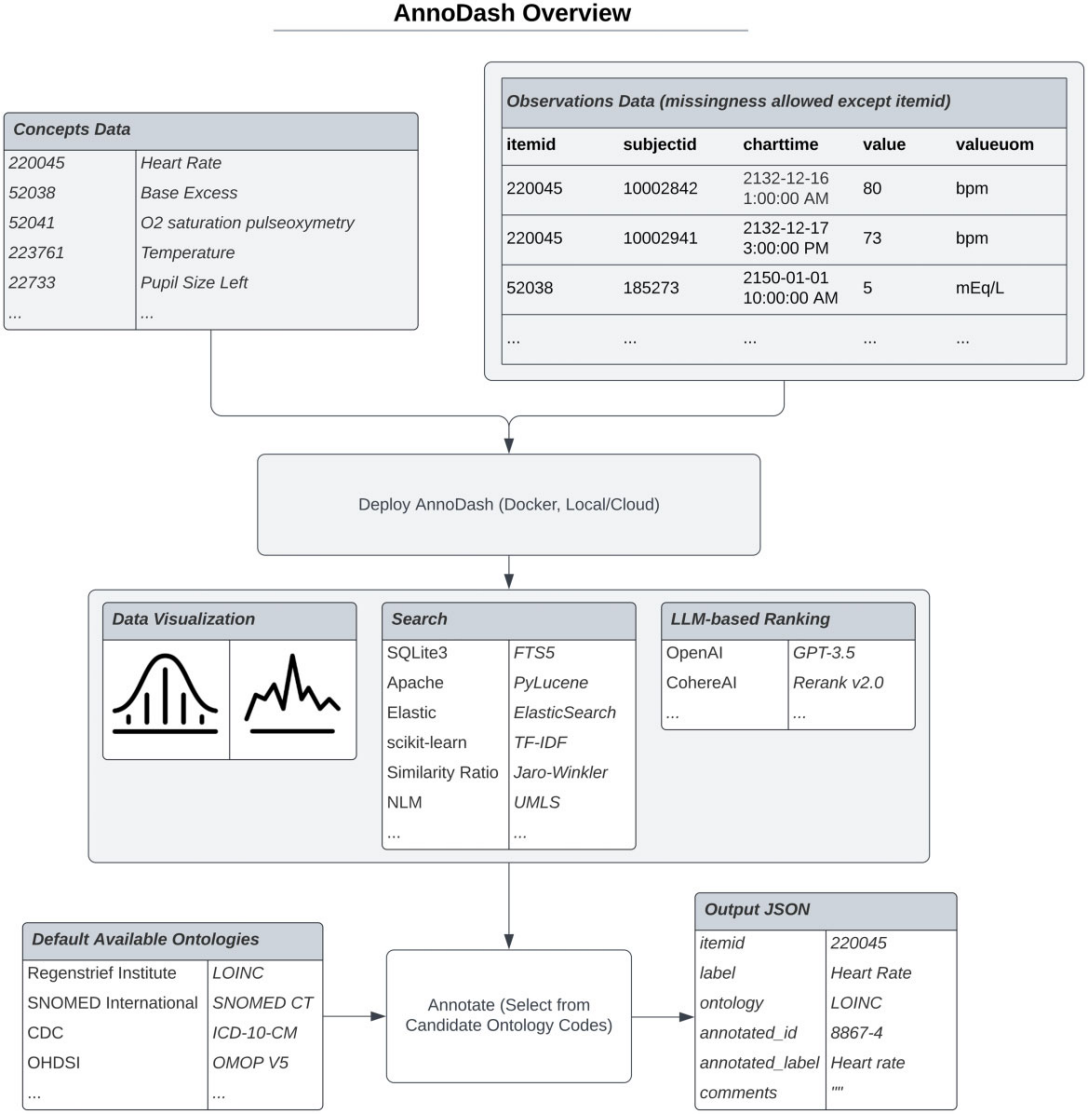
Overview diagram illustrating the AnnoDash workflow, format requirements of the data, and features for each component of the dashboard.

An overview of AnnoDash’s workflow is available in Figure 1, which clearly describes the format of the input data required and the options available for each dashboard component. Launching the dashboard requires populating a configuration file, the relevant ontology databases, and the concepts with observational data.

We evaluated the dashboard using the Medical Information Mart for Intensive Care IV (MIMIC-IV) database.^15^^,^^16^ MIMIC-IV is a large, publicly available dataset that contains deidentified health data for over 60 000 intensive care unit admissions at the Beth Israel Deaconess Medical Center. We used the 100 patient demo subset and stored laboratory and chart information in SQLite tables for evaluation. Usability testing was conducted with a small group of users with varying levels of experience in clinical medicine, technical infrastructure, and standard ontologies. Participants were asked to annotate a series of MIMIC-IV concepts using a deployed version of the dashboard. A questionnaire was then provided asking about their perceived utility of various dashboard components, as well as any additional comments. Questionnaires are included in Supplementary Table S1.

Source code for the dashboard is open-source. The application is containerized with Docker, simplifying deployment to a variety of technical infrastructure. Files for an example deployment using the MIMIC-IV demo are available on the GitHub repository.^17^ Instructions are also available for creating the ontology databases in the appropriate format, as well as for deploying the dashboard to Google Cloud Platform (GCP). Once configured and deployed, the dashboard can be used simultaneously by several users.

## RESULTS

**Figure 2.**
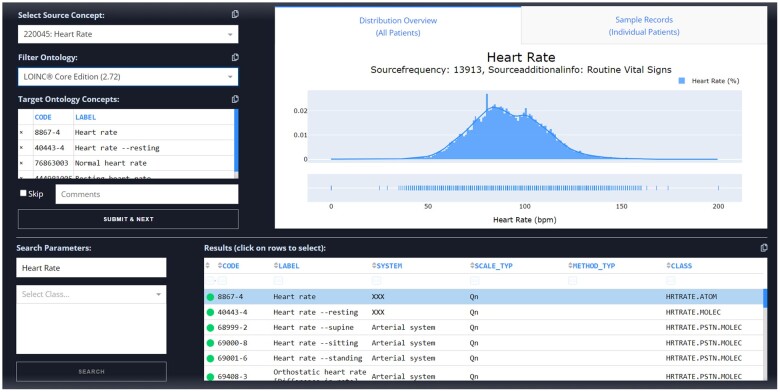
Screenshot of AnnoDash, a clinical terminology annotation dashboard.

Figure 2 shows the dashboard with the example of annotating a “heart rate” concept. As seen, the dashboard is segmented into the 3 panes. The top left pane is dedicated to the annotation functionality. The dashboard supports concept annotations to multiple ontology codes in cases where one code cannot adequately describe the entire concept. Users can do so by simply selecting (clicking) multiple codes from the search results pane (bottom pane), which would add the corresponding codes to the target ontology concepts window. The feature also allows simultaneous multi-ontology annotations, such that a concept can be annotated to codes from different systems at the same time, should the user wish to do so. This can be done by specifying the desired ontology using the appropriate dropdown and selecting codes normally.

**Figure 3.**
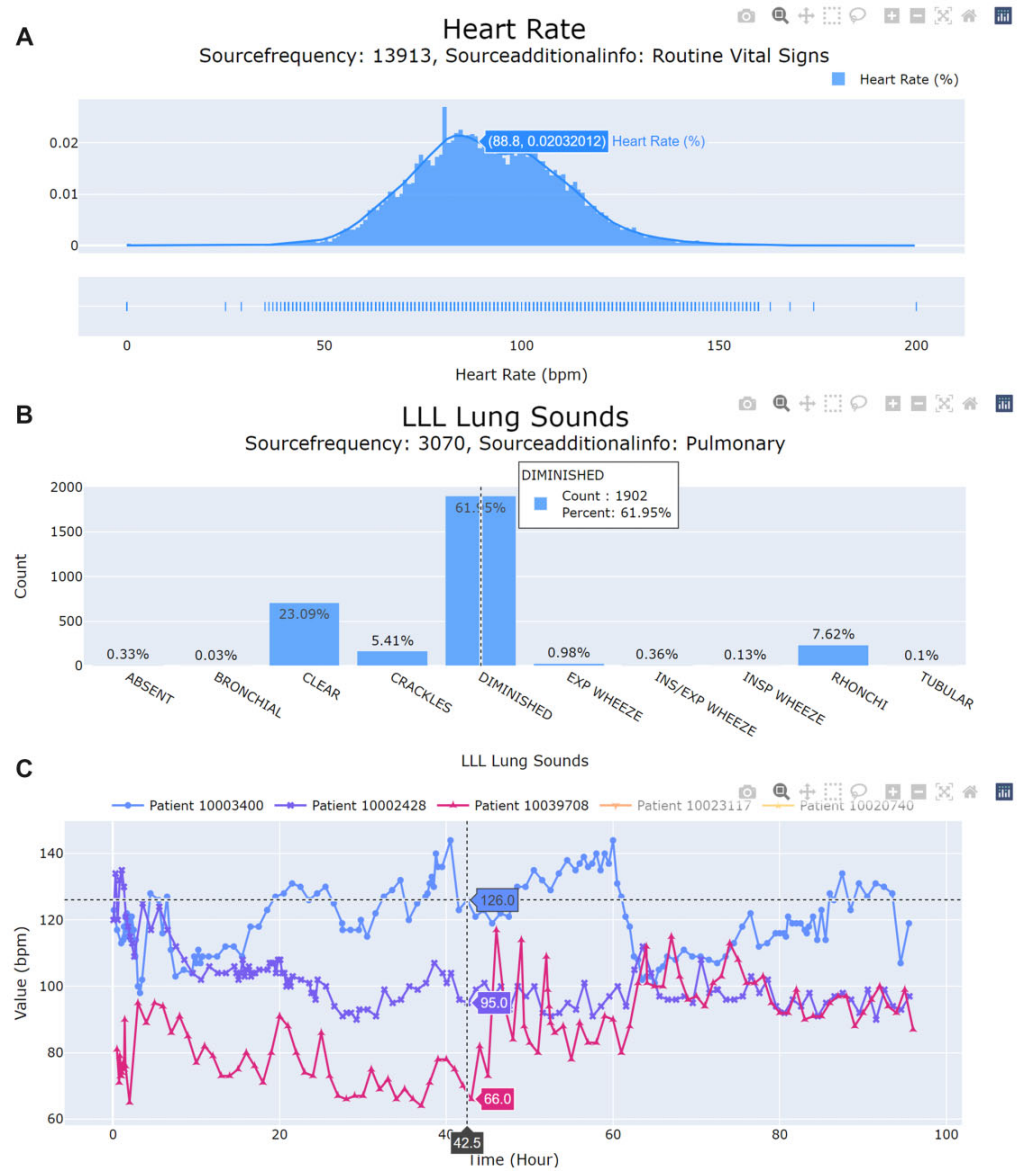
Representative data visualization plots in AnnoDash. (A) Distribution plot for the concept “heart rate“. (B) Histogram plot for the concept “left lower lobe (LLL) lung sounds”. (C) Sample time-series plot for the concept “heart rate”, visualizing temporal observations from patient records.

Figure 3 illustrates plots which exemplify the figure pane. The default tab contains a distribution of observations for numerical source concepts (Figure 3A) or a histogram of all possible observations for textual source concepts (Figure 3B). A second patient-specific tab is available to display observations for individual patient records over a 96-hour period (Figure 3C). The time window provides temporal context to the annotator, which can further increase the confidence users have in their annotations. Patients are ranked by most occurrences for a particular concept and the top 5 patients are selected automatically for plotting. Users also have the option to plot a specific patient by specifying their unique identifier.

As previously stated, these visualization modules can provide context for ambiguous concepts that would otherwise be difficult to annotate correctly. For instance, “MAP” may refer to “mean arterial pressure” or “mean airway pressure”, which can be easily disambiguated if the associated measurements are visualized. Similar plots would also help differentiate between source specimens for laboratory values.

Finally, the bottom pane is reserved for the ontology term suggestions along with metadata to help users distinguish similar ontology terms. Suggestions are automatically ranked by similarity to the textual description associated with the source concept, with scores indicated upon hovering. We compared fuzzy string matching by Jaro-Winkler distance, vector search using precomputed TF-IDF embeddings, and SQLite’s FTS5 engine coupled with Apache Lucene preprocessing and indexing. On average, suggestions for each concept took around 9 seconds for fuzzy string matching, 1.26 seconds for vector search, and 0.82 seconds for the SQLite approach. Notably, although we used established libraries for these approaches, their specific implementations have not been optimized for AnnoDash. The search functions are separated into a distinct module within the application, allowing users to provide custom plugins or modify the existing approaches to further improve suggestions.

In our pilot study of 4 users, 3 had over 3 years of experience working with medical data, and half had experience annotating medical concepts. Users required a median of 2.5 minutes to annotate a single concept during their initial exposure to AnnoDash. Participants noted difficulty annotating certain concepts if an appropriate standard term did not exist in the reference ontologies (eg, a generic concept of ectopy documentation). Technical users rated the ease of deployment as less important, whereas clinical users rated it highly. Conversely, clinical users did not consider extensibility of the dashboard as critical, whereas technical users prioritized it. All users considered displaying relevant data along with metadata for each standard ontology term as very important. Finally, both users with experience annotating medical concepts strongly favored using AnnoDash in the future. Full responses to questionnaires are available in Supplementary Table S2.

## DISCUSSION AND CONCLUSIONS

We have presented a dashboard to support the annotation of clinical concepts with one or more standard terminology systems. We believe AnnoDash provides advantages over currently available approaches. Specifically, it can circumvent Usagi’s restriction to the OMOP vocabulary by allowing users to specify the latest individual vocabularies. It also allows users to annotate concepts and terminologies within a straightforward interface supported visualizations of associated data. Suggestions are provided by natural language processing with extensible algorithms and models. The dashboard’s containerized nature allows for easy deployment in various environments, whereby a single deployment may be shared by multiple users. We hope the dashboard will facilitate interoperability and complement ongoing efforts to harmonize clinical data.

Our dashboard has limitations. It requires configuration with observed measurements for each concept using data exported from the source system, which may be nontrivial for certain hospital systems. The dashboard is also currently evaluated on the MIMIC-IV dataset, and thus its generalizability to other datasets or clinical corpora will need to be confirmed in the future. Use of string similarity approaches may result in syntactically similar but semantically distinct suggestions (eg, suggesting ventricular fibrillation for a source concept of atrial fibrillation). We aim to make additional improvements to the suggestions module to increase the likelihood of the ideal ontology code being in the top-5 of suggestions. Finally, although the quantitative measurements associated with source concepts are visualized, they are not currently used to improve suggestions without the help of LLMs. We hope to address this limitation in future work.

## Supplementary Material

ooad046_Supplementary_Data

## Data Availability

The source code and usage guide to AnnoDash are freely available at https://github.com/justin13601/AnnoDash (https://doi.org/10.5281/zenodo.8043943).^17^ The MIMIC-IV Clinical Database Demo is openly available on PhysioNet (https://doi.org/10.13026/ng9m-3n32).^16^ The full MIMIC-IV Clinical Database is available on PhysioNet (https://doi.org/10.13026/07hj-2a80).^18^
