# Supplementary material for: AnnoDash, a clinical terminology annotation dashboard
Source: JAMIA Open. 2023 Jul 8;6(3):ooad046. doi: 10.1093/jamiaopen/ooad046 (PMC10329488; doi:10.1093/jamiaopen/ooad046)
Supplement: ooad046_Supplementary_Data [file ooad046_supplementary_data.docx]

**SUPPLEMENTARY MATERIAL**

**Table S1 | AnnoDash Usability Feedback Questionnaires**

| **Introduction Questionnaires** | | | | | | | | | | |
| --- | --- | --- | --- | --- | --- | --- | --- | --- | --- | --- |
| **Q1** | How many years of experience do you have with medical data? | *None* | *<1* | | *1-2* | | *2-3* | | *3+* | |
| **Q2** | Do you have experience with annotating clinical concepts? | *No* | | | | | *Yes,* $<$*1 Year* | | *Yes,* $\geq$*1 Year* | |
| **Usability Questionnaires** | | | | | | | | | | |
|  | | *Strongly Disagree* | *Disagree* | | *Neither Disagree nor Agree* | | *Agree* | | *Strongly Agree* | |
| **Q3** | I am familiar with concepts from the **LOINC** vocabulary. |  |  | |  | |  | |  | |
| **Q4** | I am familiar with concepts from the **SNOMED CT** vocabulary. |  |  | |  | |  | |  | |
| **Q5** | I am familiar with concepts from the **ICD-10-CM** vocabulary. |  |  | |  | |  | |  | |
| **Q6** | I am familiar with concepts from the **OMOP** vocabulary. |  |  | |  | |  | |  | |
| **Q7** | I found AnnoDash to be user-friendly and accessible. |  |  | |  | |  | |  | |
| **Q8** | I found it efficient to annotate concepts using AnnoDash. |  |  | |  | |  | |  | |
| **Q9** | I would need an in-depth tutorial/guide/documentation in order to use AnnoDash. |  |  | |  | |  | |  | |
| **Q10** | I would use AnnoDash for annotating concepts in the future. |  |  | |  | |  | |  | |
|  | | | | | | | | | | |
| **Q11** | How often was your selected ontology code in the top-5 of suggestions? | *Never* | *Rarely* | | *Sometimes* | | *Often* | | *Always* | |
| **Priority Questionnaires (1 = Not at all Useful; 5 = Extremely Useful)** | | | | | | | | | | |
| **On a scale of 1-5, please rate the following priorities for an annotation tool:** | | *1* | | *2* | | *3* | | *4* | | *5* |
| **Q12** | Web-based (i.e., no installation) |  | |  | |  | |  | |  |
| **Q13** | Displaying relevant data |  | |  | |  | |  | |  |
| **Q14** | Automated concept labeling |  | |  | |  | |  | |  |
| **Q15** | Metadata of target vocabulary |  | |  | |  | |  | |  |
| **Q16** | Configurability or flexibility (i.e., plugins) |  | |  | |  | |  | |  |

**Table S2 | Responses to the Usability Feedback Questionnaires**

|  | ***Evaluator #1*** | ***Evaluator #2*** | ***Evaluator #3*** | ***Evaluator #4*** |
| --- | --- | --- | --- | --- |
| **Q1** | 3+ | 3+ | 3+ | <1 |
| **Q2** | No | Yes, $\geq$1 Year | Yes, $\geq$1 Year | No |
| **Q3** | 2 (Disagree) | 5 (Strongly Agree) | 4 (Agree) | 1 (Strongly Disagree) |
| **Q4** | 2 (Disagree) | 4 (Agree) | 4 (Agree) | 1 (Strongly Disagree) |
| **Q5** | 4 (Agree) | 5 (Strongly Agree) | 4 (Agree) | 1 (Strongly Disagree) |
| **Q6** | 2 (Disagree) | 4 (Agree) | 2 (Disagree) | 1 (Strongly Disagree) |
| **Q7** | 4 (Agree) | 5 (Strongly Agree) | 5 (Strongly Agree) | 4 (Agree) |
| **Q8** | 4 (Agree) | 5 (Strongly Agree) | 5 (Strongly Agree) | 3 (Neither Disagree nor Agree) |
| **Q9** | 2 (Disagree) | 2 (Disagree) | 3 (Neither Disagree nor Agree) | 5 (Strongly Agree) |
| **Q10** | 3 (Neither Disagree nor Agree) | 5 (Strongly Agree) | 5 (Strongly Agree) | 3 (Neither Disagree nor Agree) |
| **Q11** | 3 (Sometimes) | 4 (Often) | 4 (Often) | 3 (Sometimes) |
| **Q12** | 5 (Extremely Useful) | 5 (Extremely Useful) | 5 (Extremely Useful) | 2 (Unuseful) |
| **Q13** | 5 (Extremely Useful) | 5 (Extremely Useful) | 3 (Somewhat Useful) | 5 (Extremely Useful) |
| **Q14** | 5 (Extremely Useful) | 5 (Extremely Useful) | 5 (Extremely Useful) | 4 (Useful) |
| **Q15** | 3 (Somewhat Useful) | 5 (Extremely Useful) | 4 (Useful) | 5 (Extremely Useful) |
| **Q16** | 2 (Unuseful) | 5 (Extremely Useful) | 3 (Somewhat Useful) | 5 (Extremely Useful) |
